# Human papillomavirus vaccination in the European Union/European Economic Area and globally: a moral dilemma

**DOI:** 10.2807/1560-7917.ES.2021.26.50.2001659

**Published:** 2021-12-16

**Authors:** Edoardo Colzani, Kari Johansen, Helen Johnson, Lucia Pastore Celentano

**Affiliations:** 1 European Centre for Disease Prevention and Control, Stockholm, Sweden

**Keywords:** HPV, HPV vaccination, boys, gender-neutral, shortage

## Abstract

While many European Union/European Economic Area (EU/EEA) countries recently expanded human papillomavirus (HPV) vaccination to boys, HPV vaccine supply is currently limited for girls in low- and middle-income countries (LMIC) that are severely affected by HPV.

Globally, about 50% of countries have introduced HPV vaccination. Some LMIC with high burden of cervical cancer have not yet introduced HPV vaccination, or are reaching suboptimal vaccination coverage. While WHO issued a call for cervical cancer elimination in 2018, a global shortage of HPV vaccines is currently predicted to last at least until 2024.

We reviewed national policies of EU/EEA countries and recommendations of the World Health Organization (WHO) Strategic Advisory Group of Experts on immunisation to discuss current challenges and dose-sparing options. Several EU/EEA countries have extended HPV vaccination to boys and the European Cancer Organisation has issued a resolution for elimination of all HPV-associated cancers in both sexes. The European Centre for Disease Prevention and Control concluded in its 2020 guidance that cost-effectiveness of extending routine vaccination to boys depends on several context-specific factors. The extension of HPV vaccination to boys in EU/EEA countries may affect global availability of vaccines. Temporary dose-sparing options could be considered during the COVID-19 post-pandemic period.

## Background

Human papillomavirus (HPV) infection is the most frequent sexually transmitted disease and the second most common cause of cancer attributable to an infectious agent globally [1]. Cervical cancer is by far the most common malignancy causally associated with HPV infection. However, other less prevalent anogenital cancers, some emerging HPV-associated oropharyngeal cancers, and non-malignant but common anogenital warts are also causally associated with HPV infection and can affect both sexes [1]. Early diagnosis of HPV-associated precancerous lesions may lead to successful treatment before the development of HPV-associated cancer, yet organised screening is currently implemented and effective only against cervical cancer in women [2]. Since 2006, vaccines have been available to prevent HPV infection and HPV-associated illness. Three products are currently licensed in the European Union (EU): (i) the bivalent vaccine, Cervarix (GlaxoSmithKline Biologicals, Rixensart, Belgium); (ii) the 4-valent recombinant vaccine, Gardasil (MSD VACCINS, Lyon, France) and; (iii) the 9-valent vaccine, Gardasil 9 (MSD VACCINS).

In view of the large burden of cervical cancer attributable to HPV, immunisation strategies against HPV infection have initially focused exclusively on girls.

From the growing body of evidence, including clinical trials, field trials, large cohort studies and ecological studies on the impact of HPV vaccination, HPV vaccines have proven to be generally safe and effective against persistent HPV infection, anogenital warts, and pre-cancerous lesions, in particular when administered to HPV naive subjects. There is also evidence of substantial indirect (herd) protection when vaccine coverage is above 50% [3,4].

We review and discuss the implications of the expansion of HPV vaccination to boys in the European Union/European Economic Area (EU/EEA) in light of the current cervical cancer elimination goals and of the global shortage of HPV vaccines, which is mainly affecting girls in low- and middle-income countries (LMIC).

### Elimination goals and resolutions

Globally, by 2020, about half of all countries have introduced HPV vaccination, covering about one third of the eligible population of girls. Low- and middle-income countries have the lowest rates of introduction of HPV vaccination and some are struggling with its introduction while experiencing a high burden of cervical cancer and HPV-associated illness [5]. A recent update of the International Agency for Research on Cancer’s GLOBOCAN data showed that 84% of cervical cancer cases still occur in low-resource settings [6].

A call for elimination of cervical cancer was issued by the World Health Organization (WHO) in May 2018, followed by the American Cancer Society in June 2018 [7,8]. In September 2019, the European Cancer Organisation passed a resolution for the elimination of all HPV-associated cancers, thus setting the bar even higher [9]. Moreover, the European Commission launched Europe’s Beating Cancer Plan at the end of 2020 [10].

The WHO considers vaccination fundamental for equity in healthcare and beyond. It is one of the key elements of primary healthcare and universal healthcare coverage. The proposed Immunisation Agenda 2030 aims to “*extend the benefits of vaccines to everyone, everywhere*” and identifies vaccination ‘coverage and equity’ as one of its seven strategic priorities [11]. All vaccinations should thus be offered in an equitable way to all those that could benefit from the vaccines.

### Summary of evidence for extending routine human papillomavirus vaccination to boys in EU/EEA countries

In 2008, the European Centre for Disease Prevention and Control (ECDC) produced its first guidance on HPV vaccination in EU/EEA countries following the introduction of the first two vaccines against human papillomavirus [12]. The guidance was updated in 2012 following the collection of evidence of efficacy of HPV vaccination in males [13]. In 2020, ECDC published its first evidence-based guidance on HPV vaccination, applying the methodological guidelines of the grading of recommendations, assessment, development and evaluation (GRADE) to complement and update the information included in the previous two guidance documents [14]. This document focused on four separate topics: (i) the efficacy of the 9-valent HPV vaccine licensed by the European Medicines Agency in 2015 for males and females; (ii) the efficacy and effectiveness of any licensed HPV vaccine for males; (iii) the cost-effectiveness of extending routine HPV vaccination to males and; (iv) the efficacy and effectiveness of any licensed HPV vaccine for people living with HIV. The guidance concluded that HPV vaccination is effective in males against persistent HPV infections, anal pre-cancerous lesions and anogenital warts based on direct evidence from a pivotal 4-valent recombinant HPV vaccine trial (Table 1) and on data from immuno-bridging studies for the other licensed vaccines [14].

**Table 1:** Evidence type for efficacy of 4-valent recombinant human papillomavirus vaccination of males aged 16–26 years, summary from ECDC guidance on HPV vaccination^a^

Outcomes	Design	Vaccine efficacy		Risk of bias	Inconsistency	Indirectness	Imprecision	Evidence type (GRADE)
		%	95% CI					
HPV types 6, 11, 16 and 18								
6MPI	4-valent recombinant vaccination compared with placebo (one RCT)	85.6	73.4 to 92.9	Not serious	Not serious	Not serious	Not serious	High
AIN2/3		74.9	8.8 to 95.4	Not serious	Not serious	Not serious	Not serious	High
PeIN2/3		100.0	−3 788.2 to 100.0	Not serious	Not serious	Not serious	Very serious^b^	Low
Anogenital warts		89.4	65.5 to 97.9	Not serious	Not serious	Not serious	Not serious	High

AIN2/3: anal intraepithelial neoplasia grades 2 and 3; CI: confidence interval; ECDC: European Centre for Disease Prevention and Control; GRADE: grading of recommendations, assessment, development and evaluation; HPV: human papillomavirus; PeIN: penile intraepithelial neoplasia; RCT: randomised controlled trial; 6MPI: 6-month persistent infection.

^a^ Based on the ECDC report ‘Guidance on HPV vaccination in EU countries: focus on boys, people living with HIV and 9-valent HPV vaccine introduction’ [14].

^b^ Downgraded by one level for imprecision because of very wide 95% CI.

As part of the guidance, a systematic review of studies looking at cost-effectiveness of extending routine HPV vaccination to boys was performed (Supplementary Table) and concluded that cost-effectiveness of extending HPV vaccination to boys depended on several factors, many of which are context-specific. The cost of the HPV vaccine was the main driver of the incremental cost-effectiveness ratio (ICER) when comparing a scenario of universal vaccination with a baseline scenario of vaccination of girls only. Notably, the extension of HPV vaccination to boys appeared more cost-effective compared with increasing vaccine uptake among girls in cases where vaccination coverage among girls is persistently lower than 75–80% [15]. However, this may also depend on other factors such as public health priorities (e.g. prevention of cervical cancer) and cost of the vaccine [14]. Other factors were also able to affect the ICER, including: (i) the cost-effectiveness threshold and discount rate chosen; (ii) the selected perspective; (iii) the assumed vaccination coverage reached and maintained over time in the competing scenarios; (iv) the assumed duration of protection from the immunisation; (v) the number of HPV-associated outcomes included in the model and; (vi) the prevalence of circulating HPV serotypes and of HPV-associated illness.

### Policies on human papillomavirus vaccination in EU/EEA countries

As at December 2021, all EU/EEA countries have introduced HPV vaccination in their national programmes [16], and many countries have recently moved, or are planning to move, from a girls-only HPV vaccination strategy to a universal, or gender-neutral HPV vaccination strategy (Figure, Table 2), similar to what happened with vaccination against rubella that was initially only offered to girls and then extended to both sexes in combination with vaccination against mumps and measles [17].

**Figure:**
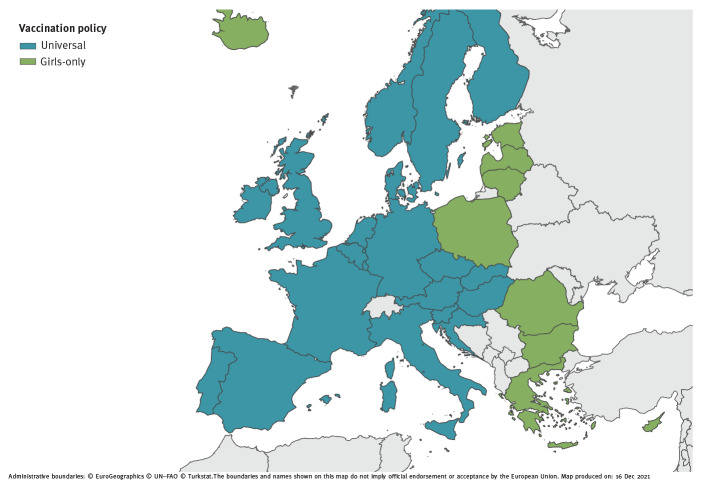
Human papillomavirus vaccination policies in EU/EEA countries and the United Kingdom, 2021

EU/EEA: European Union/European Economic Area.

**Table 2:** Overview of human papillomavirus vaccination policies in EU/EEA countries and the United Kingdom, 2007–2021^a^

Country or territory	Year of introduction	Current age targets for primary and catch-up vaccination for females and males				Delivery
		Primary vaccination (years)		Catch-up vaccination (years)		
		Female	Male	Female	Male	
Austria	2014	9	9	10–11 12–15 (PF)	10–11 12–15 (PF)	School: Grade 4 Health centre (catch-up)
The HPV vaccine has been available since February 2014 for all children in Grade 4, aged 9 years, free of charge. Before 2014, the vaccine was recommended but not publicly funded. The HPV vaccine is offered free of charge to children aged 9–12 years in public vaccination centres. Federal provinces also provide catch-up vaccinations at a reduced price for children up to the age of 15 years.						
Belgium						
Flanders	2010	13–14	13–14	12–18 (PF)	12–18 (PF)	Secondary school: Grade 1 Health centre (catch-up)
Wallonia and Brussels^b^	2011	13–14	13–14	12–18 (PF)	12–18 (PF)	Secondary school: Grade 2 Health centre (catch-up)
For girls in Flanders who do not qualify for the free vaccination or opt for a vaccine that is different from the free vaccine offered, a partial reimbursement is provided through health insurance. From September 2019, HPV vaccination is offered free of charge to all boys and girls aged 13–14 years in Flanders, Wallonia and Brussels.						
Bulgaria	2012	12–13	NA	14–26		Health centre
In 2007, an expert advisory body, including members from the Ministry of Health and the National Centre for Infectious and Parasitic Disease Control, issued official recommendations for the use of HPV vaccines for girls aged 12–18 years, before first sexual contact. In June 2009, the Ministry of Health included the HPV vaccine in the recommended vaccination list. In 2012, the National Programme for Primary Prevention of Cervical Cancer was approved. Vaccination and reimbursement of the vaccination cost by the National Health Insurance Fund for girls aged 12 years started at the beginning of 2013.						
Croatia	2016	13	13	NA		School: Grade 8
Voluntary HPV immunisation is available free of charge to all girls and boys in schools.						
Cyprus	2016	12–13	NA			School
HPV vaccination offered to girls only in schools and governmental immunisation centres since 2016.						
Czechia	2012	13–14	13–14	NA		Health centre
Since 2018, HPV vaccination of girls and boys aged 13–14 years is partially covered by public health insurance.						
Denmark	2009	12	NA	< 18	NA	Health centre
In 2014–2015, HPV vaccination was offered to girls born between 1993 and 1997. Since 2019, HPV vaccination is offered to both boys and girls.						
Estonia	2018	12–14	NA			School
From January 2020 all girls aged 12–14 years are offered the HPV vaccination within the immunisation programme.						
Finland	2013	11–12	11–12	NA		School: Grade 6–7
During the first 2 years of the programme, HPV vaccination was administered to girls aged 13–15 years while currently it is administered to girls aged 11–12 years. Boys have been offered HPV vaccination since 2020.						
France	2007	11–14 (PF)	11–14 (PF)	< 20 (PF)	< 20 (PF)	Health centre
Until September 2012, French guidelines recommended the three-dose vaccine regimen to be administered routinely to all girls aged 14 years and catch-up vaccination to women aged 15–23 years without sexual activity or with a sexual debut during the year before vaccination. In 2012, the recommendation expanded to girls aged 11–14 years with catch-up vaccination until the age of 20 years. The reimbursement rate for these vaccines is 65% of the price. Boys aged 11–14 years have been offered HPV vaccination since January 2021.						
Germany	2007	9–14	9–14	< 18	< 18	Health centre
In June 2018, the Standing Committee on Vaccination (STIKO) recommended vaccination of boys in Germany. The STIKO recommendation is needed for statutory health insurance companies to cover the costs of vaccination. Thereafter, the federal joint committee Gemeinsame Bundesausschuss decided to include HPV vaccination to all girls and boys 9–14-year-olds in the catalogue of statutory health insurance in September 2018. Since November 2018, HPV vaccination for all 9–14-year-olds, and catch-up HPV vaccination for girls and boys 15–17-year-olds, is included in the catalogue of mandatory benefits of statutory health insurance.						
Greece	2008	11–14	NA	15–18	NA	Health centre
The HPV vaccination is currently offered only to girls. Until December 2016, catch-up vaccination was offered free of charge to girls 18–26-year-olds. From January 2017 it is offered only to 15–18-year-olds. The vaccination is also recommended for 18–26-year-old men who have sex with men.						
Hungary	2014	12	12	NA		School: Grade 7
Several local governments have decided to extend the vaccine offer to those who are not eligible for the national vaccination programme because of their age. Since October 2020 HPV vaccination is also available for boys.						
Iceland	2011	12	NA			School: Grade 7
Females older than 12 years can obtain the HPV vaccine against prescription using out-of-pocket payment.						
Ireland	2010	12–13	12–13	NA		Secondary school: Grade 1
In September 2011, a catch-up programme that targeted all girls aged 17–18 years from 2011 to 2014 was introduced. Boys have been offered the vaccination since September 2019.						
Italy	2008	11	11	Variable by region	NA	Health centre
The HPV vaccination is actively offered free of charge to girls up to 12 years of age in all Italian regions. Some regions have extended the offer of vaccination to girls in other age groups. Some regions also offer free of charge HPV vaccination to people living with HIV. Most regions also consider a facilitated payment for ages not included in the primary target group. In 2015, vaccination of boys started free of charge in six regions.						
Latvia	2010	12	NA			School Health centre
The vaccination is currently offered only to girls.						
Liechtenstein	2008	11–14	11–14	15–26	15–26	NA
Liechtenstein follows the recommendations of Switzerland. Vaccination is free of charge for girls and those aged 11–16 years within the framework of the cantonal vaccination programmes. This has been extended to males aged 11–26 years since July 2016.						
Lithuania	2016	11	NA			
The HPV vaccination is currently offered only to girls.						
Luxembourg	2008	9–13	9–13	NA		Health centre
The HPV vaccination programme was introduced in 2008, targeting 12–17-year-old girls, offering bivalent or 4-valent recombinant vaccines free of charge. In 2015, the programme was changed, offering the bivalent vaccine only to 11–13-year-old girls. Since January 2019, the programme has been expanded free of charge to all boys and girls aged 9–13 years.						
Malta	2012	12	NA			Health centre
Vaccination is offered to all girls aged 12 years since 2012. One of the actions included in the national cancer plan for the Maltese islands 2017–2021 is the consolidation of the HPV vaccination programme. An evaluation of the programme will be performed at the completion of the first 5 years. This will include an exploration of the impact of expanding the programme to boys of the same age cohort of the girls already being invited.						
Netherlands	2009	12–13	12–13	NA		Health centre
In 2009, a HPV vaccination catch-up campaign was organised for girls born between 1993 and 1996 (aged 13–16 years at the time). Since 2010, girls aged 12 years are invited to receive the HPV vaccination within the National Immunisation Programme. This includes girls who were born in 1997 or later. All girls receive an invitation when turning 13 years old. Boys have been offered HPV vaccination since 2021. The vaccination is free of change and not mandatory.						
Norway	2009	12	12	NA		School: Grade 7
In 2016–2018, women born in 1991 or later were offered catch-up HPV vaccination free of charge. Since the school year 2018/19, the government offers the HPV vaccination to all boys in Grade 7 as part of the childhood immunisation programme.						
Poland	NA					
Since 2008, HPV vaccination has been recommended in the national immunisation programme for girls aged 11–12 years. The expert committee, appointed on the initiative of the Polish Paediatric Society in 2010, recommended HPV vaccines also for girls aged 13–18 years who had not been vaccinated previously. However, Poland did not introduce this vaccination into the mandatory immunisation programme. Prophylactic vaccination against HPV is charged at an extra cost in primary healthcare centres and the coverage of Polish teenagers vaccinated against HPV is estimated to be between 7.5%– 10%. Some districts have decided to introduce and finance programmes of prophylactic HPV vaccination. Currently, HPV vaccination is not part of the mandatory vaccination programme, but it is recommended for boys and girls.						
Portugal	2008	10	10	NA		Health centre
In October 2008, HPV vaccination was introduced in the national immunisation programme for girls aged 13 years born in 1995 and after. From 2009 to 2011, a catch-up vaccination campaign was run for girls aged 17 years and older born between 1992 and 1994. From 2014 to 2016, girls aged 10–13 years were covered. Since 2017, only 10-year-old girls are being vaccinated. In October 2020, the HPV vaccination was expanded to all 10-year-old children independent of sex.						
Romania	2013	11–14	NA			Health centre
In 2008, the Romanian Ministry of Health rolled out a school-based immunisation campaign providing free HPV vaccination for girls aged 10–11 years. Coverage statistics revealed that only 2.6% of the girls received vaccination and the programme was suspended. In 2009 an information campaign was launched, followed by a second vaccination programme, targeting girls aged 12–14 years. A catch-up programme was also launched, where adult women were given the opportunity to get the vaccine free of charge through their health provider. Despite the accessibility of the vaccine, uptake remained low and the school-based programme was discontinued at the end of 2011.The programme was launched for the third time in April 2013. HPV vaccination is included in the National Vaccination Programme in the category ‘Vaccination of Population at Risk’ and targets girls aged 11–14 years. The programme is not funded by the National Health System.						
Slovakia	2016	13 (PF)	13 (PF)	NA		
Neither routine HPV vaccination nor catch-up programmes have been started in Slovakia. Currently, a recommendation implemented into legislation states that if a doctor considers that there is a need for HPV vaccination, the vaccine is given to girls from the target age group. The recommendation targets other age groups who however have to pay out of pocket for the vaccine. Since January 2019, the bivalent HPV vaccine is fully reimbursed, while the 4-valent recombinant vaccine is partially reimbursed by the national healthcare system. Currently both females and males are offered the vaccination.						
Slovenia	2009	11–12	11–12	NA		School: Grade 6
The vaccination is offered to all girls and boys in Grade 6 within the compulsory national health insurance scheme. Boys are offered the HPV vaccination from the school year 2021/22.						
Spain	2007–2008	12	12	13–18	NA	School Health centre (depending on the region)
The Inter-territorial Council of the National Health System, the coordination body for the different Health services from the autonomous communities of Spain, approved a general recommendation to initiate routine HPV vaccination in Spain in 2007. The recommendation was for girls between 11 and 14 years of age, with a preference for those aged 14 years. The deadline for implementation of this recommendation was 2010. Afterwards, each autonomous community designed its own implementation programme. Three autonomous communities started in 2007 and the rest followed in 2008. Since 2015, as agreed by the Inter-territorial Council, HPV is recommended for girls aged 12 years in every region. Since 2018, HPV has also been recommended for the following risk groups/conditions: anogenital warts; hypogammaglobulinaemia; immunodeficiency and Myelokathexis (WHIM) syndrome (a primary immunodeficiency); women with solid organ and haematopoietic transplant up to 26 years of age; people living with HIV (male and female, with a three-dose schedule and up to age of 26 years); commercial sex workers up to the age of 26 years (three-dose schedule); and women with excisional treatment of the cervix. Since 2019, catch-up vaccination in girls is performed until the age of 18 years. Since 2021, HPV vaccination is offered to all girls and boys aged 12 years.						
Sweden	2012	10–12	10–12	< 18	NA	School: Grades 5–6
In 2010, the HPV vaccine was included in the free of charge national vaccination programme targeting all girls born in 1999 or later and attending Grade 5–6 in school. However, vaccinations did not start until 2012 because of delays in the procurement process. Concurrently, all counties additionally introduced free of charge catch-up vaccinations targeting girls born between 1993 and 1998. According to an update of the regulation of child vaccinations (HSLF-FS 2016:51), all girls should now be offered HPV vaccinations up to the age of 18 years. From August 2020, the vaccination is offered to all girls and boys attending school Grade 5, starting from those born in 2009.						
UK	2008–2012	11–13	11–13	< 18	NA	School: Grades 8–10 Health centre (catch-up)
Vaccination programmes and start year of the programme vary by region. Girls who initially missed HPV vaccination can receive a catch-up HPV vaccination up to the age of 18 years. At the start of the programme there was a catch-up period for girls born between 1991 and 1995. The HPV vaccination has been offered to both girls and boys since 2019.						

HIV: human immunodeficiency virus; HPV: human papillomavirus; NA: not available; PF: partially funded; UK: United Kingdom.

^a^ Funded vaccination programmes unless otherwise stated.

^b^ The regions of Brussels and Wallonia undergo the same decisions on vaccination policies.

There are several reasons that justify this decision. First, the indirect protection from vaccination of girls with suboptimal uptake, as observed in many EU/EEA countries, is in fact not sufficient to adequately protect males. Second, vaccinating only girls is not an equitable measure and does not protect men who have sex with men. Additionally, it is a less resilient strategy against sudden drops in vaccine uptake compared with a gender-neutral vaccination strategy. Finally, universal HPV vaccination is likely to be more effective and efficient in reducing HPV virus circulation in the general population even at lower levels of vaccine uptake. As at 2021, several EU/EEA countries (i.e. Austria, Belgium, Croatia, Czechia, Denmark, Finland, France, Germany, Hungary, Ireland, Italy, Liechtenstein, the Netherlands, Norway, Portugal, Slovakia, Slovenia, Spain and Sweden) and the United Kingdom (UK) have extended, or have decided to extend in the coming years, HPV vaccination to boys, while other countries are discussing the opportunity to extend it soon (Figure, Table 2).

### Human papillomavirus vaccine shortage

The WHO has highlighted that the current global HPV vaccination shortage, foreseen to last at least until 2024, might delay the introduction of HPV vaccination in the countries most in need [5]. In particular, there will not be enough vaccine doses available for girls from a number of LMIC with a high burden of cervical cancer. Extending HPV vaccination to boys implies a substantial increase in the number of doses to be procured and administered. At a time when coronavirus disease (COVID-19) vaccination may absorb large proportions of the resources available, extending HPV vaccination to boys may become complex because of logistical and acceptance challenges. Some of the potential barriers for HPV vaccination uptake in boys are parental knowledge, risk perception of HPV in men and limited recommendations from healthcare providers [18]. In 2019, there were ca 2,800,000 boys in each age cohort (aged 11–14 years) across the EU/EEA. If all EU/EEA countries expanded HPV vaccination to boys, each cohort of boys immunised would require about 2.8 million or 1.1 million extra doses of HPV vaccine at uptakes of 50% or 20%, respectively [19]. The choice of many EU/EEA countries to extend HPV vaccination to boys may thus further impact the limited supply of HPV vaccines at the global level. Moreover, it poses a moral dilemma between providing equitable access to HPV vaccines for all girls and boys within countries with well-established HPV vaccination programmes and providing equitable access to HPV vaccines for all girls globally [20].

Between 84% and 90% of all cervical cancers occur in LMIC and implementation of mass screening remains a major challenge in several of these countries. Recent projections showed that an estimated 11 million women from LMIC will be diagnosed with cervical cancer in the next 10–20 years. Many of these cervical cancers are diagnosed late and thus have poor prognosis [21]. In these countries, the prevention of persistent HPV infection through vaccination of all eligible girls could save millions of lives. In 2016, GAVI, the Vaccine Alliance, aimed to protect 40 million girls from cervical cancer, averting 900,000 deaths, through HPV vaccination by 2020. The objective was then re-adjusted to vaccination of 14 million girls and prevention of 300,000 deaths in light of the global shortage of HPV vaccines [22]. For the same reason, the recent introduction of HPV vaccination in some of the countries with the highest burden of HPV-associated illness and mortality in the world had to focus on a single cohort of girls instead of on multiple age cohorts as initially planned. Cervical cancer deaths are currently expected to reach 416,000 by 2035 without improvements in HPV prevention among girls globally [22]. Independent transmission-dynamic models have shown that high vaccination coverage of girls (> 90%) would lead to cervical cancer elimination in most LMIC by the end of this century [23].

Additionally, it should be noted that the average individual health benefit obtained from a dose of vaccine administered to a boy in the EU/EEA is substantially lower than the average individual health benefit obtained from a dose of HPV vaccine given to a girl in LMIC where cervical cancer screening is not implemented or well-established. The WHO’s Strategic Advisory Group of Experts (SAGE) on immunisation recently recommended a temporary pause in the implementation of HPV vaccination of boys in those countries that are considering doing so [24]. It was also recommended that the vaccination of girls and boys older than 15 years (i.e. catch-up vaccination strategies) and multi-age cohort vaccination strategies should temporarily pause. Meanwhile, dose-sparing options are being investigated.

### Dose-sparing options

In light of the limited evidence available, the use of a single dose of an HPV vaccine is currently not recommended. However, a substantial amount of research is ongoing. Non-randomised data from post-hoc analyses of two randomised controlled trials (RCTs) from Costa Rica and India (the latter now converted into a cohort study), using bivalent and 4-valent recombinant HPV vaccines respectively, showed that immunogenicity was still observed years after a single dose of HPV vaccination, although inferior to that observed after two or three doses [25,26]. Recent population-based data from post-licensure linkage studies from Australia, Denmark and United States (US) showed that one dose of 4-valent recombinant HPV vaccine seemed to be as effective as two or three doses to protect against high-grade cervical lesions. However, these post-licensure analyses may be subject to bias [27-29]. Five RCTs, two in Costa Rica, one in Kenya, one in Tanzania, one in the Gambia, and two observational studies in South Africa and Thailand, are currently investigating whether a single dose of HPV vaccine provides enough protection against HPV infection and HPV-associated illness. While more data from these studies are expected in the coming years, preliminary results seem promising [30].

Alternative dose-sparing options are also being considered. Vaccination schedules could be modified in order to spare doses and gain time during the current vaccine shortage. Different options were proposed at the SAGE meeting in October 2019, however, it was concluded that the feasibility of the suggested alternative schedules would need to be carefully assessed in each different context [24]. One cost-effective option proposed was to extend the interval between the first and the second dose of HPV vaccination, offering the first dose at the age of 9–10 years and the second dose at the age of 13–14 years. The success of this off-label vaccination schedule may be affected by the risk of dropouts after the first dose and the subsequent reduced uptake of the second dose. The age of initiation of sexual activity is also important to consider since HPV vaccination has shown the highest immunogenicity and efficacy in adolescents prior to the beginning of sexual life. The extension in time of the HPV vaccination schedule may lead to gaining some years before fully vaccinating the new cohorts with a second dose [24]. In the meantime, evidence of efficacy of a single HPV dose schedule would become available and global supply could be re-established so that global demand of HPV vaccines could be met.

Other gradual approaches may also be contemplated. The Immunisation Committee from the Province of Quebec in Canada recently recommended an off-label mixed schedule using the 9-valent HPV vaccine as a first dose, and the bivalent HPV vaccine as a second dose, until evidence of effectiveness of a single dose of HPV vaccine becomes available [31,32]. The rationale was based on immunological criteria with the objective of maximising the protection against HPV types 16 and 18, the two high-risk serotypes responsible for over 70% of HPV-associated cancers, while giving enough immunity against the seven additional types included in the 9-valent HPV vaccine responsible for additional HPV-associated cancers and anogenital warts. The mixed schedule was also considered the most cost-effective option to efficiently provide universal HPV vaccination. Careful and accurate monitoring of HPV-associated infections and illnesses (e.g. anogenital warts) was recommended in order to confirm the soundness and continuation of this off-label vaccination strategy [31].

The COVID-19 pandemic has affected national routine immunisations and may influence the future priorities of vaccination programmes in many countries. A modelling study estimated that, because of the impact of the pandemic on HPV vaccine uptake, over 130,000 cases of genital warts and between 2,882 and 6,487 cases of cervical cancer will not be avoided in the US over the next 100 years, with over 60% of them occurring in the next 50 years [33].

### Conclusions

Too many girls living in LMIC with a high burden of cervical cancer are not vaccinated against HPV, hence the global call for action for cervical cancer elimination. In high-income countries, including several EU/EEA countries, the priority has shifted towards the prevention of all HPV-associated illness and the equity of access to HPV vaccination for all genders thanks to successful vaccination and screening strategies against cervical cancer. In view of the current global HPV vaccines shortage which could be affected by the current COVID-19 pandemic in different ways, and given the growing global demand for HPV vaccines, there are different aspects to consider including equity of benefit, equity of access globally, current and future vaccine supply, national and international health needs and priorities, costs and political imperatives. The SAGE issued clear and sensible temporary recommendations, however, countries that have already extended HPV vaccination to boys, or have already decided to extend it, may face important challenges to provisionally reverting or modifying their decision after long debates on the opportunity to do so.

The dose-sparing options suggested by SAGE represent possible temporary solutions, in particular the extension of the time interval between the first and the second dose of the HPV vaccine. However, the communication and implementation of such temporary decisions may be difficult and might affect HPV vaccination confidence and uptake of the full two-dose schedule. The potential impact of the COVID-19 pandemic on supply and uptake of other vaccines suggests postponing any further change of the current vaccination schedules to the post-pandemic phase.

## Supplementary Data

194000 Supplement
