# Supplementary material for: Human papillomavirus vaccination in the European Union/European Economic Area and globally: a moral dilemma
Source: Euro Surveill. 2021 Dec 16;26(50):2001659. doi: 10.2807/1560-7917.ES.2021.26.50.2001659 (PMC8728487; doi:10.2807/1560-7917.ES.2021.26.50.2001659)
Supplement: Supplement [file 20-01659_COLZANI_SUPPLEMENT.pdf]

This supplementary material is hosted by *Eurosurveillance* as supporting information alongside the article “Human papillomavirus vaccination in the European Union/European Economic Area and globally: a moral dilemma” on behalf of the authors who remain responsible for the accuracy and appropriateness of the content. The same standards for ethics, copyright, attributions and permissions as for the article apply. Supplements are not edited by Eurosurveillance and the journal is not responsible for the maintenance of any links or email addresses provided therein

TABLE. Main characteristics of studies on cost-effectiveness analysis of universal human papillomavirus vaccination, 2004–2017 (n = 21)

Author	Publication year	Country	Currency	Analysis year	Horizon (years)	Perspective	Vaccine used	Vaccine schedule	Health outcome unit	CEA threshold defined
Taira et al. [19]	2004	US	USD	2001	38	TPP	2-valent	Three doses	QALYg	50,000–100,000
Elbasha et al. [15]	2007	US	USD	2005	100	TPP	4-valent recombinant	Three doses	QALYg	No
Kulasingam et al. [8]	2007	Australia	AUD	2005	73	TPP	Bivalent	Three doses	QALYg	No
Jit et al. [12]	2008	UK	GBP	2006	100	TPP	4-valent recombinant	Three doses	QALYg	30,000
Kim et al. [20]	2009	US	USD	2006	100	SP	4-valent recombinant	Three doses	QALYg	50,000
Zechmeister et al. [13]	2009	Austria	EUR	2007	52 and 80	TPP and SP	Bivalent	Three doses	LYg	No
Olsen et al. [10]	2010	Denmark	EUR	2007	62	TPP	4-valent recombinant	Three doses	QALYg	No
Elbasha et al. [14]	2010	US	USD	2008	100	TPP	4-valent recombinant	Three doses	QALYg	50,000–100,000
Chesson et al. [16]	2011	US	USD	2008	100	SP	4-valent recombinant	Three doses	QALYg	100,000
Burger et al. [18]	2014	Norway	USD	2010	100	SP	4-valent recombinant	Three and two doses	QALYg	83,000
Laprise et al. [9]	2014	Canada	CAD	2010	70	TPP	4-valent recombinant	Three and two doses	QALYg	40,000
Pearson et al. [11]	2014	New Zealand	NZD	2011	98	TPP	4-valent recombinant	Three doses	QALYg	45,000
Bresse et al. [1]	2014	Austria	EUR	2012	100	TPP	4-valent recombinant	Three doses	QALYg	No
Blakely et al. [2]	2014	New Zealand	NZD	2011	98	TPP	4-valent recombinant	Three doses	QALYg	No
Haeussler et al. [3]	2015	Italy	EUR	2015	Long-term	TPP	4-valent recombinant	Three doses	QALYg	25,000–40,000
Jiménez et	2015	Norway	NOK	2014	100	TPP and SP	4-valent	Three doses	QALYg	215,000

al. [7]							recombinant and bivalent			
Olsen et al. [17]	2015	Denmark	EUR	2008	62 and 40	TPP	4-valent recombinant	Three and two doses	QALYg	No
Qendri et al. [5]	2017	Netherlands	EUR	2011	Lifetim e	TPP	Bivalent	Two doses	LYsg	40,000
Damm et al. [4]	2017	Germany	EUR	2010	100	TPP and SP	4-valent recombinant and bivalent	Three and two doses	QALYg	50,000
Largeron et al. [21]	2017	Germany	EUR	2014	100	TPP	4-valent recombinant and 9-valent	Two doses	QALYg	40,000
Mennini et al. [6]	2017	Italy	EUR	2014	100	TPP	4-valent recombinant and 9-valent	Two doses	QALYg	25,000–40,000

References:

[1] Bresse X, Goergen C, Prager B, Joura E. Universal vaccination with the quadrivalent HPV vaccine in Austria: impact on virus circulation, public health and cost-effectiveness analysis. Expert Rev Pharmacoecon Outcomes Res. 2014;14(2):269-81.

[2] Blakely T, Kvizhinadze G, Karvonen T, Pearson AL, Smith M, Wilson N. Cost-effectiveness and equity impacts of three HPV vaccination programmes for school-aged girls in New Zealand. Vaccine. 2014;32(22):2645-56.

[3] Haeussler K, Marcellusi A, Mennini FS, Favato G, Picardo M, Garganese G, et al. Cost-Effectiveness Analysis of Universal Human Papillomavirus Vaccination Using a Dynamic Bayesian Methodology: The BEST II Study. Value Health. 2015;18(8):956-68.

[4] Damm O, Horn J, Mikolajczyk RT, Kretzschmar MEE, Kaufmann AM, Delere Y, et al. Cost-effectiveness of human papillomavirus vaccination in Germany. Cost Eff Resour Alloc. 2017;15:18.

[5] Qendri V, Bogaards JA, Berkhof J. Health and Economic Impact of a Tender-Based, Sex-Neutral Human Papillomavirus 16/18 Vaccination Program in the Netherlands. J Infect Dis. 2017;216(2):210-9.

[6] Mennini FS, Bonanni P, Bianic F, de Waure C, Baio G, Plazzotta G, et al. Cost-effectiveness analysis of the nine-valent HPV vaccine in Italy. Cost Eff Resour Alloc. 2017;15:11.

[7] Jimenez E, et al. Cost-Effectiveness of HPV-Vaccination of Boys Aged 12 in a Norwegian Setting Oslo: Norwegian Centre for the Health Services at The Norwegian Institute of Public Health (NIPH); 2015 [Available from: http://www.ncbi.nlm.nih.gov/books/NBK390581/.

[8] Kulasingam S, Connelly L, Conway E, Hocking JS, Myers E, Regan DG, et al. A cost-effectiveness analysis of adding a human papillomavirus vaccine to the Australian National Cervical Cancer Screening Program. Sex Health. 2007;4(3):165-75.

[9] Laprise JF, Drolet M, Boily MC, Jit M, Sauvageau C, Franco EL, et al. Comparing the cost-effectiveness of two- and threedose schedules of human papillomavirus vaccination: a transmission-dynamic modelling study. Vaccine. 2014;32(44):5845-53.

[10] Olsen J, Jepsen MR. Human papillomavirus transmission and cost-effectiveness of introducing quadrivalent HPV vaccination in Denmark. Int J Technol Assess Health Care. 2010;26(2):183-91.

[11] Pearson AL, Kvizhinadze G, Wilson N, Smith M, Canfell K, Blakely T. Is expanding HPV vaccination programs to include school-aged boys likely to be value-for-money: a cost-utility analysis in a country with an existing school-girl program. BMC Infect Dis. 2014;14:351.

[12] Jit M, Choi YH, Edmunds WJ. Economic evaluation of human papillomavirus vaccination in the United Kingdom. BMJ. 2008;337:a769.

[13] Zechmeister I, Blasio BF, Garnett G, Neilson AR, Siebert U. Cost-effectiveness analysis of human papillomavirusvaccination programs to prevent cervical cancer in Austria. Vaccine. 2009;27(37):5133-41.

[14] Elbasha EH, Dasbach EJ. Impact of vaccinating boys and men against HPV in the United States. Vaccine. 2010;28(42):6858-67.

[15] Elbasha EH, Dasbach EJ, Insinga RP. Model for assessing human papillomavirus vaccination strategies. Emerg Infect Dis. 2007;13(1):28-41.

[16] Chesson HW, Ekwueme DU, Saraiya M, Dunne EF, Markowitz LE. The cost-effectiveness of male HPV vaccination in the United States. Vaccine. 2011;29(46):8443-50.

[17] Olsen J, Jorgensen TR. Revisiting the cost-effectiveness of universal HPV-vaccination in Denmark accounting for all potentially vaccine preventable HPV-related diseases in males and females. Cost Eff Resour Alloc. 2015;13:4.

[18] Burger EA, Sy S, Nygard M, Kristiansen IS, Kim JJ. Prevention of HPV-related cancers in Norway: cost-effectiveness of expanding the HPV vaccination program to include pre-adolescent boys. PLoS One. 2014;9(3):e89974.

[19] Taira AV, Neukermans CP, Sanders GD. Evaluating human papillomavirus vaccination programs. Emerg Infect Dis. 2004;10(11):1915-23.

[20] Kim JJ, Goldie SJ. Cost effectiveness analysis of including boys in a human papillomavirus vaccination programme in the United States. BMJ. 2009;339:b3884.

[21] Largeron N, Petry KU, Jacob J, Bianic F, Anger D, Uhart M. An estimate of the public health impact and cost-effectiveness of universal vaccination with a 9-valent HPV vaccine in Germany. Expert Rev Pharmacoecon Outcomes Res. 2017 Feb;17(1):85-98.
